# Formation of self-assembled gold nanoparticle supercrystals with facet-dependent surface plasmonic coupling

**DOI:** 10.1038/s41467-018-04801-9

**Published:** 2018-06-18

**Authors:** Kaifu Bian, Hattie Schunk, Dongmei Ye, Austin Hwang, Ting Shan Luk, Ruipeng Li, Zhongwu Wang, Hongyou Fan

**Affiliations:** 1 Sandia National Laboratories, Albuquerque, NM 87123 USA; 2 NSLS II, Brookhaven National Laboratories, Upton, NY 11973 USA; 3 Cornell High Energy Synchrotron Source, Ithaca, NY 14853 USA; 4 Department of Chemical and Biological Engineering, University of New Mexico, Albuquerque, NM 87106 USA

## Abstract

Metallic nanoparticles, such as gold and silver nanoparticles, can self-assemble into highly ordered arrays known as supercrystals for potential applications in areas such as optics, electronics, and sensor platforms. Here we report the formation of self-assembled 3D faceted gold nanoparticle supercrystals with controlled nanoparticle packing and unique facet-dependent optical property by using a binary solvent diffusion method. The nanoparticle packing structures from specific facets of the supercrystals are characterized by small/wide-angle X-ray scattering for detailed reconstruction of nanoparticle translation and shape orientation from mesometric to atomic levels within the supercrystals. We discover that the binary diffusion results in hexagonal close packed supercrystals whose size and quality are determined by initial nanoparticle concentration and diffusion speed. The supercrystal solids display unique facet-dependent surface plasmonic and surface-enhanced Raman characteristics. The ease of the growth of large supercrystal solids facilitates essential correlation between structure and property of nanoparticle solids for practical integrations.

## Abstract (web summary)

Macroscopically large supercrystals are very difficult to assemble from metallic nanoparticles. Here, the authors use a binary solvent diffusion method to form sub-millimeter gold nanoparticle supercrystals with rare *hcp* symmetry, and discover that they exhibit facet-dependent optical properties.

## Introduction

Metallic and semiconductor nanoparticles (NPs) have been widely researched in terms of their ability to self-assemble into ordered supercrystals (SCs)^1–7^. These SCs displayed not only the intrinsic characteristics which belong to individual NP building blocks but also unique collective optical, electronic, and mechanical properties which are tunable by the mesostructure^8,9^. The self-assembly of NPs is a complex process which involves numerous interactions including van der Waals attraction, Coulombic and magnetic forces, steric repulsion, and capillary forces etc.^10–12^. By tuning these interactions, the SC morphology could be controlled^1,7,13–15^. Highly ordered single SCs are potentially the key to understand complex chemical and physical processes such as optoelectronic coupling, surface plasmon-based sensing, and pressure-induced interparticle coalescence^11,12,16–18^. Previously, the largest SCs were reported to be only tens of micrometers^4,10,19,20^. To explore the structure–property relationship which connects nanomaterials to practical applications, it is important to obtain SCs large enough to be systematically manipulated and analyzed by a variety of characterization methods. Large defect-free SC is also critical for device integration.

In this work, SCs with sub-millimeter size are prepared from dodecanethiol-capped spherical gold NPs by a counter-diffusion method. The NP solution is slowly driven to supersaturation by increasing anti-solvent concentration, resulting in heterogeneous SC growth. The structure in the SCs is characterized by a recently developed supercrystallography technique based on small- and wide-angle X-ray scattering (SAXS and WAXS) diffractometry^4^. The NPs formed hexagonal close packed (*hcp*) symmetry as confirmed by SAXS patterns, while isotropic interactions between the NPs are revealed by WAXS measurements. The preference of *hcp* over face-centered cubic (*fcc*) superlattice is attributed to the differences in free energy between tetrahedral and octahedral voids in the stacking hexagonal monolayers. Through combined simulation and controlled kinetic investigation, we discover that the size and quality of the SCs are determined by initial NP concentration and diffusion speed. These SCs are large enough to be readily manipulated and characterized by techniques designed for macroscopic specimens. Optical spectroscopy results reveal strong surface-enhanced Raman scattering (SERS) effect from the SC solids and unique facet-dependent plasmonic resonance.

## Results

### Gold supercrystal growth

**Fig. 1:**
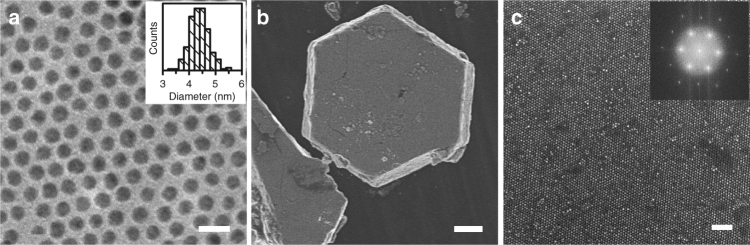
Electron microscopy characterizations of gold NPs and SCs. **a** TEM image of the synthesized gold NPs. Scale bar is 10 nm. Inset shows statistics of the particle diameter. **b** SEM image of a SC. Scale bar is 5 μm. **c** High-resolution SEM image of the top surface of the SC showing hexagonal packing. Scale bar is 50 nm. Inset shows the corresponding FFT pattern

Dodecanethiol-capped gold NPs were synthesized using a one-step method and dispersed in toluene^21^. As shown by Transmission electron microscopy (TEM) image (Fig. 1a) the product spherical gold NPs had an average diameter of 4.4 nm and standard deviation of 8%. In the early stage of the experiment, SCs were grown by a counter-diffusion method that has been reported to produce SCs of various NP species^2,19,22,23^. Briefly, anti-solvent isopropanol (IPA) was added on top of toluene solution of gold NPs forming a liquid–liquid interface. The solvents were then allowed to diffuse into each other. As IPA concentration increased in the NP phase, gold NPs became oversaturated and slowly precipitated. The process took approximately 1 week. More experimental details are provided in Methods. The product SCs displayed highly faceted hexagonal disk shape and size up to tens of micrometers (Fig. 1b). Such morphology suggested a hexagonal packing of the constituent NPs. It was confirmed by high-resolution scanning electron microscopy (SEM) image of the SC surface (Fig. 1c), which revealed a nearly perfect hexagonal close-packing array. The corresponding fast Fourier transform (FFT) pattern showing clear second-order peaks evidenced the long-range translational ordering and suggested a single SC, i.e., each grain contained only one crystal domain.

### Structural characterization by X-ray scattering supercrystolography

**Fig. 2:**
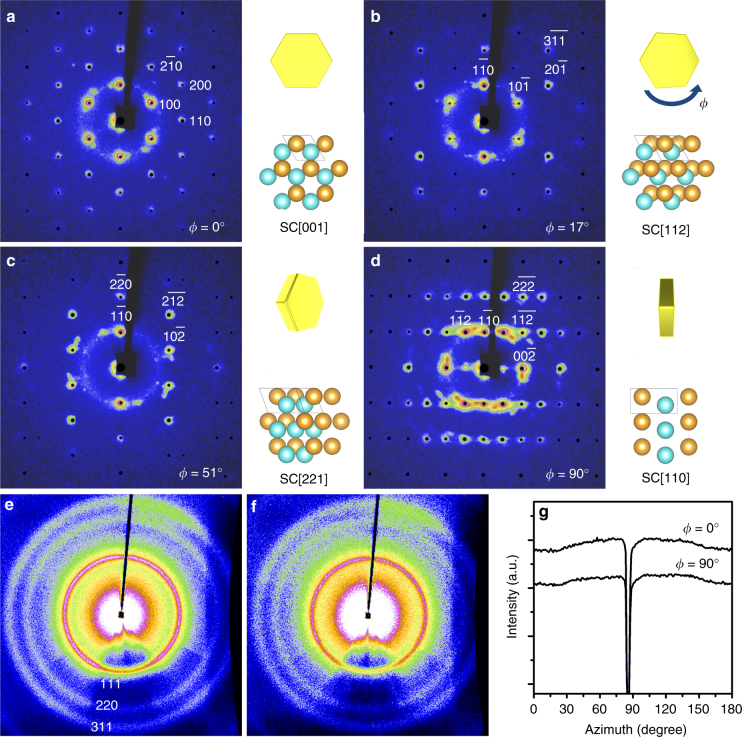
Supercrystallography analysis of a single gold SC at varying rotational angle phi (*ϕ*). **a**–**d** SAXS patterns in selected projections. Simulated peaks (black dots) from an *hcp* superlattice are overlaid on top of experimental patterns with Miller indices labeled. Insets of **a**–**d** present corresponding schematic illustrations of a rotating SC with X-ray beam shooting perpendicular to paper and an *hcp* superlattice in the same projections as labeled by SC[hkl]. Three consecutive hexagonal monolayers are shown in two different colors to emphasize the ABA packing for visual aid. WAXS patterns from the same SC with **e** *ϕ* = 0° and **f** *ϕ* = 90°, collected simultaneously with the SAXS patterns in **a**, **d**, respectively. Powder scattering rings from gold atomic lattice are marked with Miller indices. **g** Integrated azimuthal WAXS spectra of the Au (111) peak. The sharp dips were caused by beam-stop blockage

To decode the three-dimensional (3D) structure in the gold SCs, they were characterized by a recently developed supercrystallography technique^4^. Comprehensive sets of SAXS and WAXS images were collected from a SC rotating around one of its high symmetry axes *ϕ*. The abundant SAXS and WAXS data were analyzed to provide structure information at meso- and atomic scales respectively. Figure 2a–d shows the representative SAXS patterns. Sharp and multiple-ordered peaks confirmed long-range translational order, consistent with the results from SEM images. These peaks were indexed to a single *hcp* superlattice (insets of Fig. 2a–d). An additional set of SAXS patterns (Supplementary Fig. 1) were collected from the same SC with a different rotation axis 30° apart to unambiguously confirm the symmetry. The *hcp* lattice parameters were measured as *a* = *b* = 6.7 nm, *c* *=* 10.9 nm, and *α* = *β* = 120°, *γ* = 90°. The separation between nearest neighbor NPs was 2.3 nm, shorter than twice the length of a free dodecanethiol ligand (1.8 nm). It indicated interdigitation between ligands as to lower enthalpy via ligand–ligand van der Waals attraction^1,5,24^. With such short interparticle separation, the interstitial voids in the *hcp* superlattice were mostly occupied by the ligands. In addition to translational symmetry, the orientation of the gold NPs in the SC was simultaneously measured by WAXS. Figure 2e, f shows the WAXS patterns 90° apart. In both cases, as clarified by the integrated scattering intensity as a function of azimuthal angle (Fig. 2g), continuous powder scattering rings were observed, indicating randomly oriented NPs. The lack of orientational order suggested isotropic interparticle interactions, which agrees with the spherical particle shape.

### Influences of nanoparticle concentration and diffusion speed

**Fig. 3:**
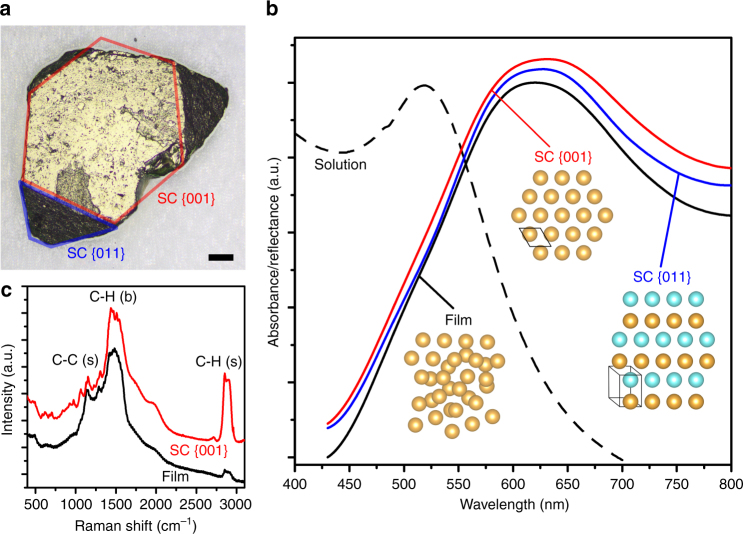
Large gold SC of sub-millimeter size and optical characteristics. **a** Photograph of a gold SC measured 490 μm. Scale bar is 50 μm. The blue and red frame outline SC {011} and SC {001} surface, respectively. **b** Optical reflectance spectra (normalized) collected from two different facets (blue and red) of the SC and a drop-cast film (black solid line) and absorption from the NP solution (black dashed line). **c** Anti-Stokes Raman spectra of dodecanethiol ligand collected from the surface of SC (red) and film (black). The peaks are labeled with corresponding vibration modes with (s) = stretching and (b) = bending

In order to optimize the diffusion-induced growth and further improve the size and quality of the gold SCs, a series of crystallization was conducted under various conditions. The influence of initial NP concentration *C*_NP,0_ was explored by growing SCs from solutions with *C*_NP,0_ *=* 2, 4 and 8 mg/mL with results summarized by Supplementary Fig. 2a–c. As *C*_NP,0_ increased from 2 to 8 mg/mL, the average size was increased and the uniformity of the SCs was improved as well. In addition, the size of the largest observed SCs doubled from ~23 μm to ~41 μm. It can be intuitively explained by the fact that higher *C*_NP,0_ provided more NPs as building materials for larger SCs. The effect of diffusion speed was also investigated by doubling the height of the liquid column to slow down the counter-diffusion process from approximately 1 week to 1 month. In this case *C*_NP,0_ was maintained at 8 mg/mL for consistency. A representative photograph of the product SCs is presented in Supplementary Fig. 2d showing that slow diffusion resulted in noticeably larger SCs. The average SC size increased from 23 μm to 37 μm while the maximum almost doubled from 41 μm to 79 μm. It is worth pointing out that the total quantity of the gold NPs in this scenario was doubled due to increased volume of the NP solution. Assuming that the diffusion speed was irrelevant to the SC growth, an average SC size of 23 μm × ^3^√2 = 29 μm was expected, 28% smaller than the experimental value of 37 μm. Therefore, it can be concluded that lower diffusion speed contributed to larger SCs in addition to *C*_NP,0_. Upon identification of the two key factors determining the size and quality of SCs, the growth was further optimized to be carried out in a capillary tube and with a very high *C*_NP,0_ = 25 mg/mL. The capillary tube, with an inner diameter of only 2 mm, significantly elongated the diffusion duration from c.a. 1 month to 3 months due to enhanced surface tension/boundary effect and minimized perturbation. Gold SCs with well-defined facets and a record large size up to 0.5 mm, equivalent to ~7.5 × 10^4^ unit cells, were obtained (Fig. 3a).

### Surface plasmon resonance in sub-millimeter gold supercrystals

Large SCs enabled easy transfer, manipulation, and characterizations and thus opened a new door to the study of collective properties of the NPs in the ordered arrays. Optical reflectance spectra were collected from two different facets of the SC shown in Fig. 3a and compared to that from a drop-casted gold NP film as a reference to explore the relationship between NP packing structure and surface plasmon behavior. The reference film was prepared by drop-casting gold NPs on a glass slide then dried quickly and possessed an amorphous mesostructure as confirmed by the SAXS pattern in Supplementary Fig. 3. As summarized by Fig. 3b and Table 1, the plasmon resonance peak shifted between samples indicating different degrees of coupling effect. As another demonstration Raman spectrum of the dodecanethiol ligand was collected from the SC {001} surface and compared to the reference film (Fig. 3c). Three major vibration modes were identified including C-C stretching (1145 cm^−1^), C-H bending (1475 cm^−1^), and C-H stretching (2880 cm^−1^). The absence of S-C stretching (~650 cm^−1^) and S-H stretching (~2580 cm^−1^) peaks confirmed grafting of dodecanethiols on the gold NP surface. It was also observed that the C-H (s) peak was significantly strengthened at the SC surface due to SERS effect, by approximately 9 times with respect to the film reference. This peak at 2880 cm^−1^ corresponds to an anti-Stokes scattered photon with absolute energy of 1.95 eV or 635 nm (shifted from 780 nm incident photon) which was in close vicinity of the plasmonic peak at 631.5 nm of SC {001}. The improved SERS was attributed to strongly coupled electric field by the highly ordered SC. It is worth pointing out that due to the large size of the SCs, the measurements were performed on a widely available Raman spectrometer designed for macroscopic samples, facilitating future development of quicker and cheaper SERS-based characterization and detection technologies.

**Table 1:** Plasmon peak position and width of gold SC

Sample	Peak center (nm)	HWHM (nm)
Solution	528.7	63.5
Film	620.4	73.4
SC {011}	627.3	73.6
SC {001}	631.5	83.0

## Discussion

**Fig. 4:**
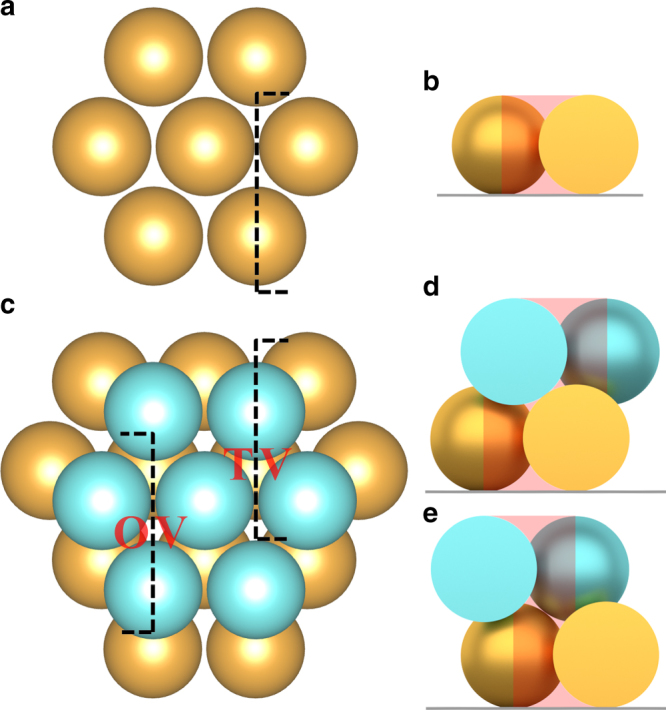
Schematic illustration of the early stage of the SC growth. **a** The first hexagonal monolayer of NPs. **b** Cross-section as marked by dashed line in **a**. **c** Two consecutive monolayers and cross-sections showing **d** a TV and **e** an OV. The red shades outline the free space in these voids

The combined results of electron microscopy and X-ray scattering confirmed the successful growth of record large gold SCs of sub-millimeter size with *hcp* mesostructure. The 3D hexagonal packing of spherical particles can be achieved by either *fcc* or *hcp* lattice. Both consist of the identical hexagonal monolayers with the only difference in stacking arrangement of ABCA vs. ABAB. For hard spheres, *fcc* offers a slightly higher translational entropy than *hcp* by a margin of only ~0.001*k*_B_*T* per particle^25–27^, too small to dominantly affect packing morphology. Therefore, a random *hcp* configuration, e.g. ABACB, is often observed with colloidal particles. In this study, gold NP assembled into *hcp* SCs exclusively. It was attributed to the role of ligands that had been reported to be critical in determining structures in NP superlattices^7,10,13,14^. The disk shape of SCs and their preference to attach to a substrate suggested a heterogeneous layer-by-layer growth. The SC formation began with a first hexagonal monolayer (golden spheres in Fig. 4a, b) that then served as the foundation for further growth. The second NP layer (blue spheres) prefer to attach on top of the first layer at the identical triangular void sites to maximize nearest neighbor contacts due to strong ligand–ligand attractions. When adding the third layer, as illustrated by Fig. 4c–e, there were two options, namely tetragonal voids (TVs) and octahedral voids (OVs), leading to *hcp* and *fcc*, respectively. Two reasons could have contributed to the dominance of TV sites in the gold SCs. On one hand, as the NP continued to stack into the *hcp* superlattice, the OVs connected to form tunnels penetrating the entire SC. These tunnels provided space for ligand and solvent molecules, resulting in higher entropy in them^28^. On the other hand, the relatively hollow OV tunnels tolerated more anti-solvent molecules than the TV sites which were mostly occupied by ligand molecules. Therefore, an incoming NP would avoid the thermodynamically less preferred OVs according to Flory–Huggins theory and attach to the TVs to minimize energy by ligand interdigitation.

In this study, the gold NP solution was driven to a state of supersaturation by counter-diffusion and then the free energy gained was released by the formation of precipitate^29^. The size and quality of SCs were also found to relate to the initial NP concentration *C*_NP,0_ and the diffusion speed. To obtain insights, the solubility of NPs and the diffusion process were quantitatively analyzed. The solubility of gold NPs *C*_NP_ in toluene/IPA mixture was experimentally measured as a function of the volume fraction of IPA, *x*_IPA_ (Supplementary Fig. 4b, details in Methods). For visual aid and computational convenience, the data were fitted to an exponential solubility curve $C_{{\mathrm{NP}}} = 2.0{\mathrm e}^{ - 3.9x_{{\mathrm{IPA}}}} - 0.040$. This curve divided the phase diagram into two regimes. In regime S, the NPs are soluble, and precipitate when the system entered regime P. Such a phase diagram served as an important guide for the optimization of SC growth. The usage of this phase diagram is illustrated by the examples in Supplementary Fig. 4c–e. Higher *C*_NP,0_ results in larger enclosed area in the diagram meaning greater quantity of NP precipitation and larger SCs.

**Fig. 5:**
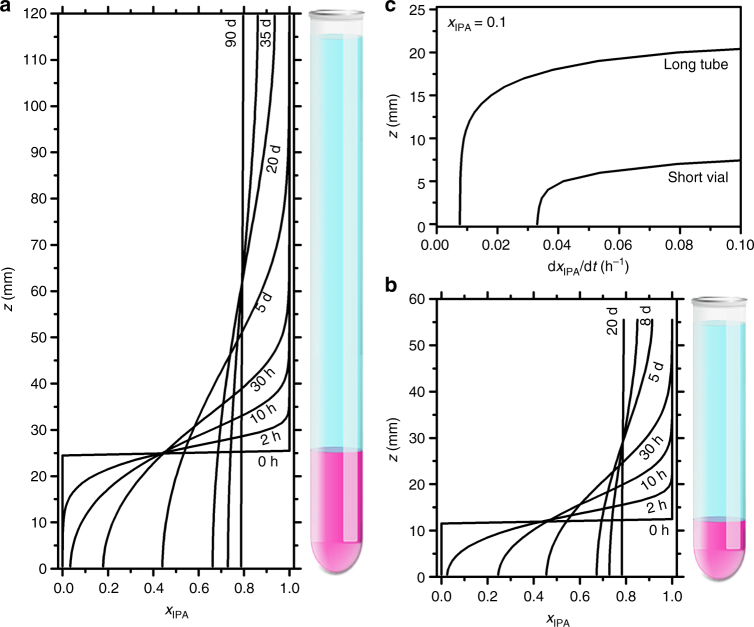
Simulated solvent composition during counter-diffusion. **a** Slow and **b** fast diffusion. The corresponding schematics on the side illustrate the initial configuration of the liquid column (purple: gold NPs in toluene; blue: IPA). **c** Spatial distribution of diffusion speed when *x*_IPA_ reached 0.1

To understand how diffusion speed influenced the SC size, the counter-diffusion process was simulated by a two-solvent finite element model. Figure 5a, b shows the evolution of mixture composition *x*_IPA_ over time for the slow and fast diffusion scenarios, respectively. Three trends consistent with experiments were observed. (1) In both cases, the initial sharp interface gradually blurred. Eventually the systems became a homogeneous solution. (2) The time elapsed in the slow diffusion was approximately four times of the fast case. (3) Slow diffusion displayed a gentler concentration gradient, i.e. $\partial x_{{\mathrm{IPA}}}/\partial z$, which contributed to larger SC sizes by limiting nuclei formation. To quantify the diffusion speed, $\left. {\partial x_{{\mathrm{IPA}}}/\partial t} \right|_{x_{{\mathrm{IPA}}} = 0.1}$ was tracked. According to the aforementioned phase diagram, with *C*_NP,0_ = 8 mg/mL, more than 80% of NPs already precipitated when *x*_IPA_ increased to 0.1. Therefore, this early stage is crucial to the size and quality of SC. On one hand, Fig. 5c showed a nearly four times lower speed of composition variation in the slow diffusion case which enabled longer relaxation time for NP crystallization. On the other hand, slow diffusion resulted in a taller bottom region (10 mm vs. 5 mm) with low diffusion speed, providing extra space for large SC formation. It also explained the observation that most SCs were harvested near the bottom of the test tubes.

As a remarkable consequence of the achievement of sub-millimeter-sized SCs, optical characterization was performed on individual SC facets. As shown by Fig. 3b and Table 1 the plasmonic peak red-shifted from 529 nm to ~620 nm upon precipitation from solution indicating interparticle coupling induced by significantly reduced interparticle distance^30,31^. A closer examination revealed that the SC sample displayed a further red-shift from the amorphous film by 7–11 nm as well as broadened peak width. Even more interestingly, a slight difference was identified between two adjacent SC facets which had not been observed before to the best of our knowledge. We determined that the strength of coupling effect followed the order: film < SC {011} < SC {001} which was attributed to their different surface morphology. Enhancement of coupling is expected for highly ordered arrays over amorphous stacking of NPs^32^. Additionally, the spatial arrangement of NPs at SC surface could further affect coupling. As illustrated by the insets of Fig. 3b, a gold NP at the SC {001} facets has 6 nearest neighbors while only 5 for SC {011}. In addition, the higher degree of symmetry of SC {001} could lead to emergence of extra coupling modes which broadened the resonance spectrum, analogous to the mini-band formation in quantum dots^33^. Due to the different surface patterns, the NP packing density at SC {011} is 7.2% lower than SC {001} which increased the average interparticle separation from 2.3 nm to 2.5 nm. Such increment had been reported to cause a 2.0% shift of plasmon resonance energy in two-dimensional (2D) hexagonal lattice of large (10.5 nm) gold NPs^30^, comparable to the 0.7% observed in this work. As another factor, the perfect planar SC {001} was expected to provide stronger 2D in-plane coupling than the slightly wavy surface of SC {011}. Such facet-dependent optical property showed that it is more appropriate to treat SCs as anisotropic media and use tensors for their mathematic descriptions rather than scalars, an excellent analogy to traditional crystal optics.

## Methods

### Synthesis of gold nanoparticles

Spherical gold NPs were synthesized using a one-step, oil-phase method^21^. Briefly, the synthesis was carried out in air by mixing the metal precursor, AuPPh_3_Cl, with dodecanethiol as capping ligand in toluene. The reducing agent, tert-butylamine-borane complex, was then added. The mixture was left to stir for 24 h at room temperature. The raw product, which was dark purple in color, was washed twice in ethanol, filtered and redispersed in toluene for characterization. TEM images indicate that the gold NPs were monodisperse and 4.4 ± 0.4 nm in diameter.

### Supercrystal growth

The crystallization of gold NPs took place in vertically positioned glass test tubes (~13 cm). In a typical growth, a test tube was first filled with colloidal solutions of gold NPs in toluene of varying concentrations. The anti-solvent IPA was then slowly added on top with a volumetric ratio of IPA/toluene = 4:1. An interface between the two solvents was formed. The total height of the liquid was either ~56 or 120 mm for fast and slow diffusion, respectively. The tubes were left undisturbed as the counter-diffusion proceeded, indicated by the blurring interface. The growth was considered complete when the interface disappeared and the mixture became homogeneous and colorless. The product SCs were collected by removing the liquid and rinsed by and then stored in ethanol.

### Gold nanoparticle solubility in binary solvent system

An excess amount of dried gold NPs was dissolved in toluene/IPA mixtures with varying composition, reported by volume fraction of IPA, *x*_IPA_. The mixture was sonicated to aid dissolution and then centrifuged to obtain saturated NP supernatant which was then transferred to a quartz cuvette for optical absorption measurements. Dilution was needed for high concentration samples. The gold NP displayed a purple color in solution and a surface plasmon resonance peak at ~520 nm. This peak was used to determine nanoparticle concentration via Beer–Lambert law. Figure 4a shows selected absorption spectra of saturate gold NP solutions with different toluene/IPA compositions. As expected, absorbance decreased as the anti-solvent fraction increased and NP solubility reduced.

### Simulation of counter-diffusion

The counter-diffusion was simulated by a two-solvent one-dimensional diffusion finite element model. Tyn–Calus method and Chevron mixing rule were used to calculate the average binary diffusivity in the toluene/IPA mixture^34,35^. In a typical simulation, a toluene column at bottom and an IPA column on top were initiated with a height ratio of 1:4. The total height of liquid was set to either 56 mm or 120 mm to simulate the fast and slow diffusion scenarios, respectively. Then, the liquid column was divided into 100 elements. The composition change over time in each element was calculated according to Fick’s law at a time interval of 1 min. The simulation was stopped when the composition difference between the top and bottom elements became less than 1%.

### Characterization methods

SAXS and WAXS data were collected at B1 station of Cornell High Energy Synchrotron Source (CHESS) with a monochromatic beam of wavelength 0.04859 nm. The beam was collimated to 100 μm in diameter. The sample-detector distance was calibrated using CeO_2_ and Silver behenate standards. The scattering patterns were collected by a large-area Mar345 2D detector. In a typical measurement, a piece of gold SC was mounted on a two-circle diffractometer which enables rotation in two axes *χ* and *ϕ* (insets of Fig. 2a–d). First, a high symmetry orientation of the supercrystal was located by trial measurements. The angle *χ* was adjusted so that the *ϕ* axial of the diffractometer was parallel to one of the high symmetry axes. Then, the SC was rotated around *ϕ* axial for 180° while SAXS and WAXS patterns were collected at an interval of 1°. The X-ray scattering data were interpreted by comparison to simulated diffraction patterns generated by the commercial software Crystal Maker (version 9.2.2) to determine the mesostructure. TEM images of the gold NPs were captured by a JEOL 2010 TEM operated at 200 kV. The average size was determined by statistics of NPs in multiple images. SEM measurements were performed on a Hitachi S-5200 microscope operated at 5–15 kV. Optical microscopy photographs were captured with an Olympus BX51 microscope equipped with 10×, 20×, or 100× objective lenses, by an Olympus UC50 camera. Optical absorption spectra of NP solution samples were measured using a Perkin Elmer Lambda 950 spectrophotometer. Reflectance spectra from SC and film surfaces were measured by a custom-built optical system. The white light source from a xenon arc lamp (Energetiq EQ99X) was delivered to the sample using a multimode optical fiber and a 10× microscope objective. The beam was converged to a c.a. 40 μm spot, as calibrated by a pin hole, small enough to map individual SC facets. The reflected light was collected with a beam splitter and delivered to an Acton SP2300i spectrometer. A bare glass slide was used as reference. The reported data were averaged spectra from multiple locations on a given SC facet. Raman spectroscopy was carried out in a Thermo Scientific DXR smart Raman spectrometer equipped with a universal platform sampling accessory and a 780 nm laser of 50 μm beam size.

### Data availability

The data that support the findings of this study are available from the corresponding author upon reasonable request.

## Electronic supplementary material

Supplementary Information
