# Supplementary material for: Formation of self-assembled gold nanoparticle supercrystals with facet-dependent surface plasmonic coupling
Source: Nat Commun. 2018 Jun 18;9:2365. doi: 10.1038/s41467-018-04801-9 (PMC6006263; doi:10.1038/s41467-018-04801-9)
Supplement: Supplementary file 1 — Supplementary Information [file 41467_2018_4801_MOESM1_ESM.pdf]

Supplementary Information

Formation of Self-Assembled Gold Nanoparticle Supercrystals with Facet-Dependent Surface Plasmonic Coupling

Kaifu Bian[1], Hattie Schunk[1], Dongmei Ye[1], Austin Hwang[1], Ting Shan Luk[1], Ruipeng Li[2], Zhongwu Wang[3], Hongyou Fan[1,4,*]

[1]Sandia National Laboratories, Albuquerque, NM 87123, United States;

[2]NSLS II, Brookhaven National Laboratories, Upton, NY 11973, United States;

[3]Cornell High Energy Synchrotron Source, Ithaca, NY 14853, United States;

[4]Department of chemical and Biological Engineering, University of New Mexico, Albuquerque, NM 87106, United States.

*Corresponding author: hfan@sandia.gov

List of Supplementary Figures:

Figure 1. Supercrystallography analysis of a single gold SC with a different rotation axis.

Figure 2. Gold SCs grown under different conditions.

Figure 3. SAXS patterns of the reference gold NP film showing an amorphous mesostructure.

Figure 4. Phase diagram of solubility of gold NP in toluene-IPA solvent mixture.

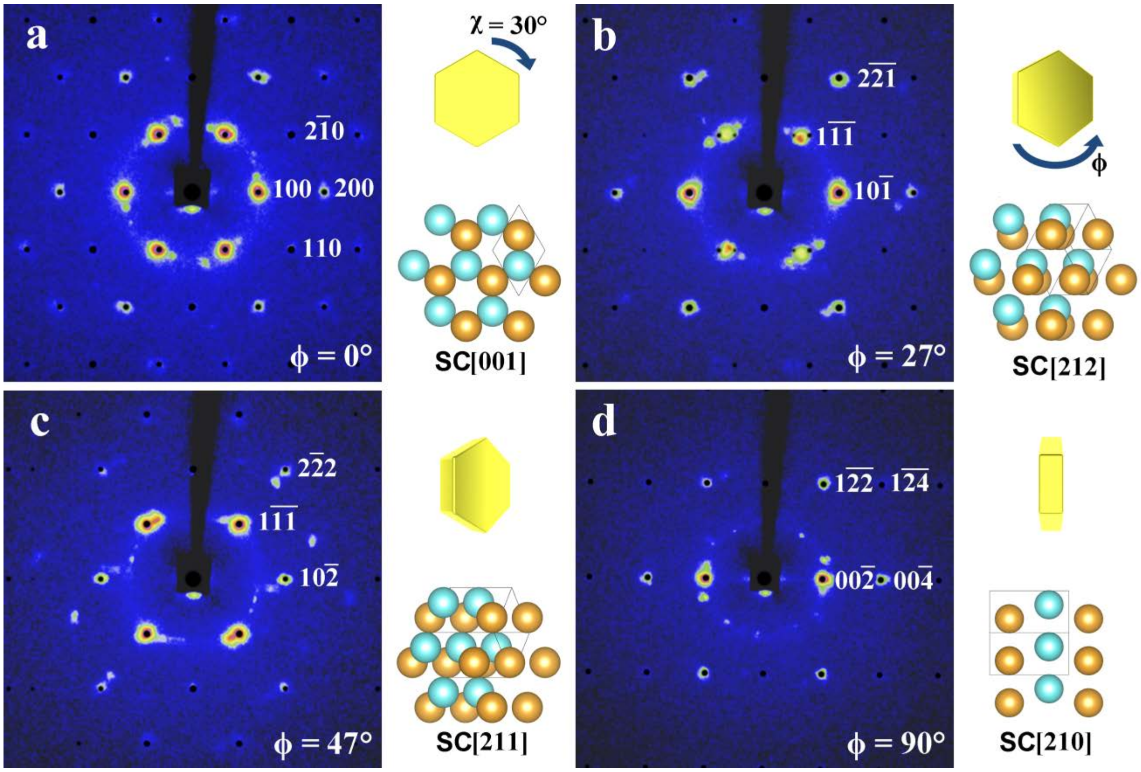

Figure 1. Supercrystallography analysis of a single gold SC with a different rotation axis, χ = 30° (a)-(d) SAXS patterns in selected projections. Insets are schematic illustrations of a rotating SC with X-ray beam shooting into paper. And an *hcp* superlattice in the corresponding projections as labeled by superlattice [hkl]. Three hexagonal layers are shown in two different colors to emphasize the ABAB packing for visual aid.

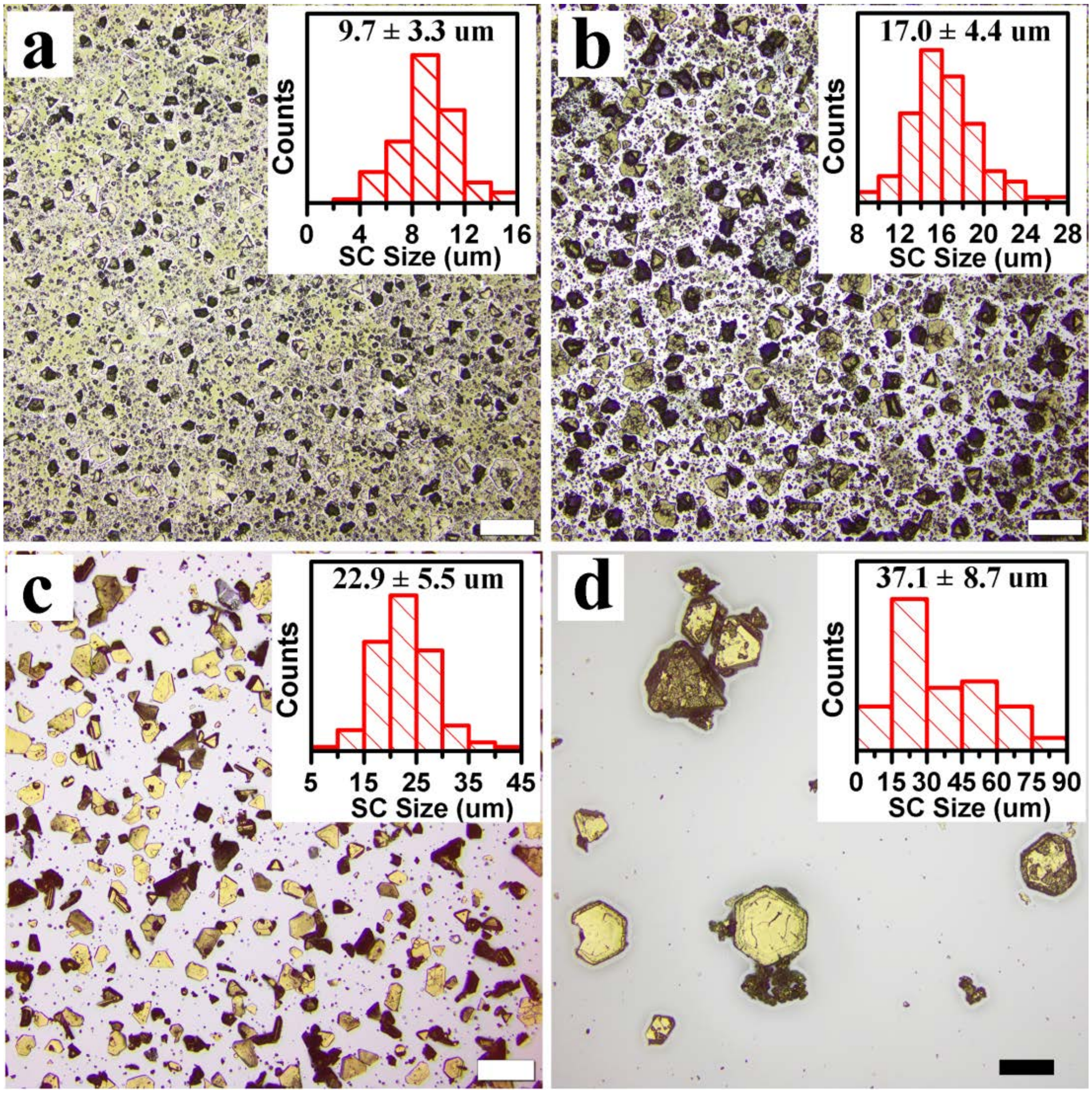

Figure 2. Gold SCs grown under different conditions. Fast diffusion with different initial nanoparticle concentration of (a) 2, (b) 4, and (c) 8 mg/mL and (d) slow diffusion. Insets are statistical histograms of corresponding SC sizes. Scale bars are 50 μm.

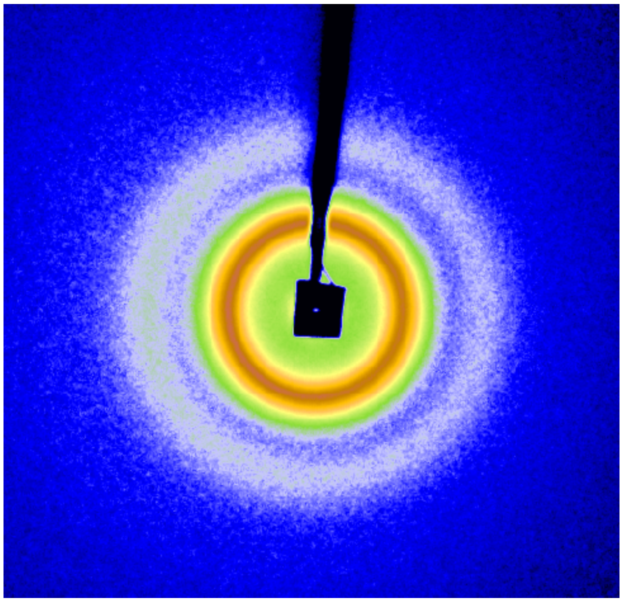

Figure 3. SAXS patterns of the drop-cast reference gold NP film showing an amorphous mesostructure.

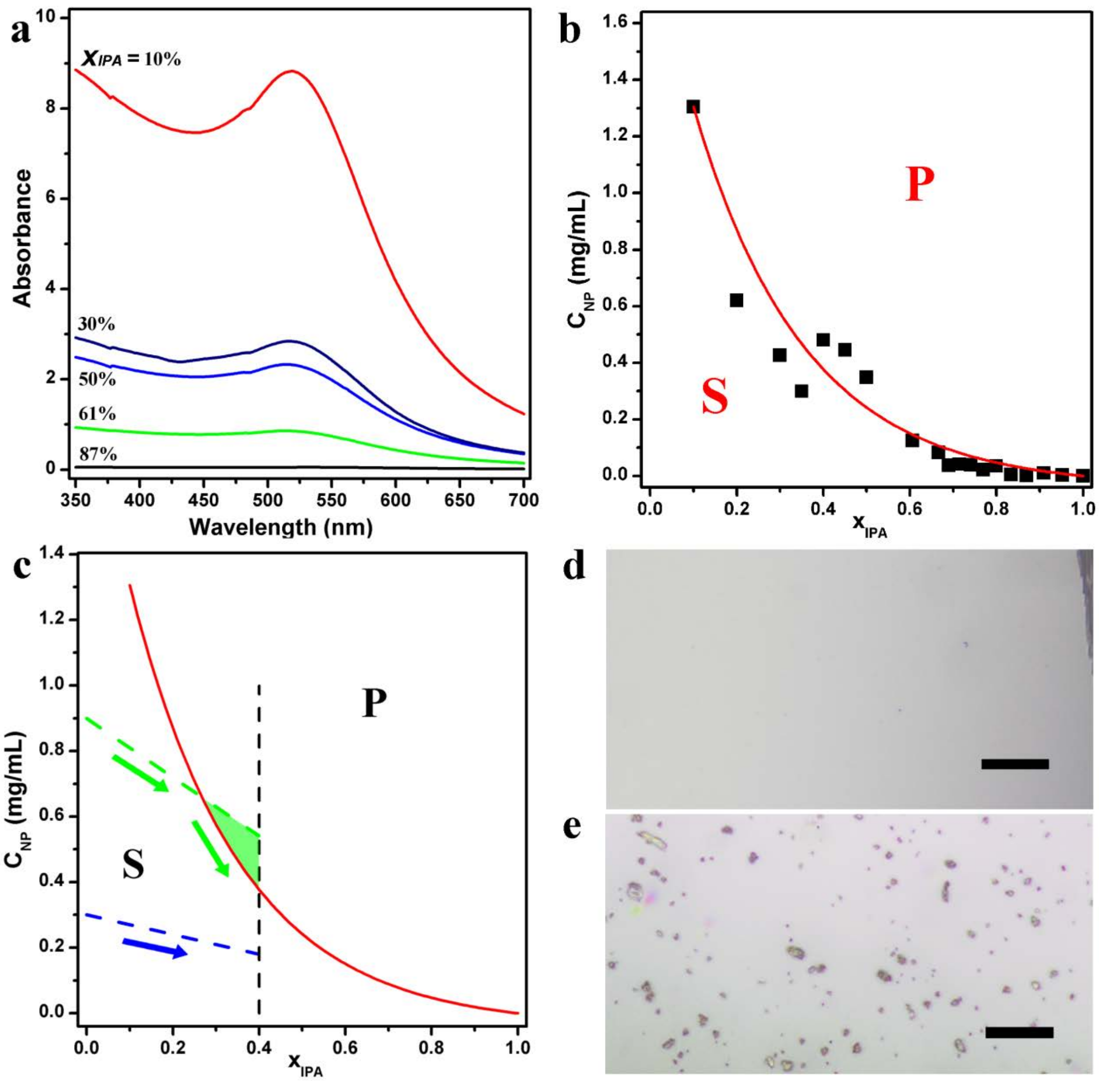

Figure 4. Phase diagram of solubility of gold NP in toluene-IPA solvent mixture. (a) Selected optical absorption spectra of saturated solution of gold NPs in varying volume fraction of IPA, x_{IPA}. (b) Experimentally measured (squares) and fitted (solid line) solubility curve as a function of. This phase diagram was tested by a pair of experiments with initial NP concentration $C_{NP,0}$ of 0.3 and 0.9 mg/mL. (c) shows the dilution paths of gold solution in the two experiments. In the case of lower $C_{NP,0}$ (blue path), the system remained in regime S and no precipitation was expected. In contrast, in the higher concentration case the dilution line (green path) intercepted the solubility curve and entered regime P to predict precipitation of quantity proportional to the enclosed area (green shade). These predictions were then confirmed by optical microscopy images. No precipitation was observed in the 0.3 mg/mL case (d) while the substrate in the 0.9 mg/mL case displayed grains of gold SC (e). These grains had size of ~ 1 µm and could play the role of nucleus for further supercrystal growth. Scale bars are 10 µm. The feature at the top right corner of (d) is the edge of the substrate, as a visual aid to indentify the focal plane.
